# Intrinsically disordered regions that drive phase separation form a robustly distinct protein class

**DOI:** 10.1016/j.jbc.2022.102801

**Published:** 2022-12-14

**Authors:** Ayyam Y. Ibrahim, Nathan P. Khaodeuanepheng, Dhanush L. Amarasekara, John J. Correia, Karen A. Lewis, Nicholas C. Fitzkee, Loren E. Hough, Steven T. Whitten

**Affiliations:** 1 Department of Chemistry and Biochemistry, Texas State University, San Marcos, Texas, USA; 2 Department of Chemistry, Mississippi State University, Mississippi, USA; 3 Department of Cell and Molecular Biology, University of Mississippi Medical Center, Jackson, Mississippi, USA; 4 Department of Physics, University of Colorado Boulder, Boulder, Colorado, USA; 5 BioFrontiers Institute, University of Colorado Boulder, Boulder, Colorado, USA

**Keywords:** intrinsically disordered protein, protein self-assembly, protein-protein interaction, protein sequence, subcellular organelle, AUC, area under the curve, BMRB, Biological Magnetic Resonance Bank, ID, intrinsically disordered, IDP, intrinsically disordered protein, IDR, intrinsically disordered region, PCA, principal component analysis, PDB, Protein Data Bank, PS, phase-separating

## Abstract

Protein phase separation is thought to be a primary driving force for the formation of membrane-less organelles, which control a wide range of biological functions from stress response to ribosome biogenesis. Among phase-separating (PS) proteins, many have intrinsically disordered regions (IDRs) that are needed for phase separation to occur. Accurate identification of IDRs that drive phase separation is important for testing the underlying mechanisms of phase separation, identifying biological processes that rely on phase separation, and designing sequences that modulate phase separation. To identify IDRs that drive phase separation, we first curated datasets of folded, ID, and PS ID sequences. We then used these sequence sets to examine how broadly existing amino acid property scales can be used to distinguish between the three classes of protein regions. We found that there are robust property differences between the classes and, consequently, that numerous combinations of amino acid property scales can be used to make robust predictions of protein phase separation. This result indicates that multiple, redundant mechanisms contribute to the formation of phase-separated droplets from IDRs. The top-performing scales were used to further optimize our previously developed predictor of PS IDRs, ParSe. We then modified ParSe to account for interactions between amino acids and obtained reasonable predictive power for mutations that have been designed to test the role of amino acid interactions in driving protein phase separation. Collectively, our findings provide further insight into the classification of IDRs and the elements involved in protein phase separation.

Many intracellular reactions occur within membrane-free compartments that form spontaneously from the cellular milieu (1). Examples of such compartments include P-bodies, Cajal bodies, the nucleolus, paraspeckles, and germ granules (2, 3, 4). The formation of membrane-less organelles is facilitated primarily, though not exclusively (5, 6), by proteins that are intrinsically disordered (ID) or contain large ID regions (IDRs), collectively termed intrinsically disordered proteins (IDPs) (4, 7). Because these protein-rich droplets typically exist in dynamic, liquid-like states rather than as fixed complexes (1, 2), this transition is referred to as liquid-liquid phase separation or, more generally, protein phase separation. By forming specific compartments and micro-environments, protein phase separation exerts control over the spatial organization and biochemical reactivity within cells (8, 9). Indeed, protein phase separation has been found to modulate chemical and biochemical reactions (10, 11, 12) and its dysregulation has been associated with several human diseases (13, 14, 15).

Due to the critical role of protein phase separation in cell function and disease, significant efforts have been made to determine the physical mechanisms responsible for phase separation behavior. Mutation and sequence analysis have implicated cation-π, π-π, π/sp^2^, and hydrophobic interactions, inferred in part by the prevalence of both hydrophobic amino acids and particular combinations of amino acids (*e.g.*, arginine and tyrosine) within phase-separating (PS) IDRs (16, 17, 18, 19, 20, 21, 22). Groups of amino acids driving cohesive interactions are often characterized as “stickers” and are frequently interspaced with small polar residues acting as “spacers” (22, 23, 24, 25). In addition, charge composition and patterning appear to contribute to the regulation of phase separation by IDRs (20, 26, 27, 28, 29). Successfully predicting the relationship between primary sequence and phase separation behavior is key to understanding the underlying molecular mechanisms and identifying the cellular processes that rely on protein phase separation. Effective predictive algorithms might also reveal how mutations affect phase separation–associated disease states.

Several methods have been developed to predict which protein sequences drive phase separation (30, 31). Algorithms including PSPredictor and PSPer are based on the composition of databases of proteins that are known to phase separate (28, 32). Other predictors aim to classify proteins based on specific subgroups with similar behavior, such as PLAAC for prions (33), catGRANULE for ID and RNA binding ability (34), and CRAPome that scores protein–protein interactions (35). While these other predictors were not originally engineered to predict phase separation per se, they have been used as proxies for potential phase separation behavior (31, 36, 37). Uniquely, PScore was developed based on a specific mechanism thought to drive phase separation: the propensity of cation-π and π-π interactions to drive cohesive protein interactions (16, 38). Simulation models of IDRs have also been used to identify which protein domains drive phase separation as well as how mutations will affect phase separation behavior of those proteins (39, 40, 41, 42, 43). The diversity of successful approaches for predicting protein phase separation indicates that multiple complementary mechanisms are responsible for this phenomenon.

We previously developed a predictive model of protein phase separation, ParSe (“Partition Sequence”), that identifies PS IDRs starting from predictions of hydrodynamic size, which is indicative of the relative strength of intramolecular as compared to solvent interactions (44). The core assumption of ParSe is that intramolecular cohesion that compacts monomeric proteins is correlated with intermolecular cohesion that drives phase separation (45, 46, 47, 48). ParSe uses a sequence-based model of the polymer scaling exponent, *v*_*model*_ (49, 50), which was originally developed from polymer theories to extract information on the balance of self and solvent interactions in long homopolymers (51, 52). When *v*_*model*_ is combined with a second sequence-based parameter, the intrinsic propensity for a sequence to form β-turns (53), the algorithm can distinguish between sequences belonging to one of three classes of protein regions: folded, ID, and PS ID (44). We proposed a physical mechanism whereby transient β-turn structures reduce the desolvation penalty of forming a protein-rich phase and increase exposure of atoms involved in π/sp^2^ valence electron interactions. In this mechanism, β-turns could promote energetically favorable cohesion points and act as stickers in a stickers and spacer model of protein phase separation. This role as stickers potentially explains the observed higher propensity for turns in IDRs that drive phase separation *in vivo* (44, 53).

However, the prior study did not test whether the combination of *v*_*model*_ and β-turn propensity was uniquely able to distinguish folded, ID, and PS ID sequences, as would be required if this putative mechanism is necessary for phase separation. In the current study, we first curated the sequence training sets to expand the folded and ID categories. Our curated list of proteins that are ID but not thought to drive phase separation acts as a key negative control, enabling us to distinguish which features of IDRs in particular drive protein phase separation (31). Using the expanded sequence sets, we exhaustively tested all amino acid property scales found in the Amino Acid Index Database (54) for their ability to separate folded, ID, and PS ID sequences. We show that the three sequence sets are distinct in their means when quantified by the majority of amino acid scales, revealing that there are robust property differences between biologically relevant ID and PS ID sequences, not unlike the differences between folded and ID sequences. Thus, although phase separation is a physical process resulting from a balance between the solvent and the macromolecule, it appears that biological phase separation occurs in solvent conditions similar enough that this class of sequences can be identified irrespective of the details of the cellular states that drive phase separation.

We applied principal component analysis (PCA) to identify the extent of variability between our sequence sets and the optimal combinations of property scales that maximize the distinction between ID and PS ID sequences. The resulting predictor, ParSe version 2 (v2), uses sequence hydrophobicity to distinguish folded from ID and, subsequently, *v*_*model*_ and a conformational parameter to distinguish ID from PS ID. In general, PS ID sequences exhibit enriched β-turn and depleted α-helix propensities. ParSe v2 more accurately predicts these regions from the amino acid sequence than the original version. We then compared our predicted propensity for protein phase separation with experimental results on mutant sequences designed to test the role of π- and charge-based interactions in phase separation behavior. We found that only by including effects representing interactions between amino acids could we accurately predict phase separation behavior of these mutants. Given the high fidelity of ParSe even in the absence of these interaction terms, it appears there are multiple diverse mechanisms that can drive protein phase separation and that PS ID sequences can be robustly identified through simple combinations of amino acid property scales.

## Results

### Construction of protein sequence datasets

A limitation of the previous work, including our own (44), has been the relatively small set of sequences used to train predictors. We first sought to alleviate this problem by identifying additional sequences in our two negative control categories, folded proteins and IDRs, which are not thought to phase separate. The importance of well-defined negative control sets has been highlighted recently by Pansca *et al* (31) and Cai *et al* (55). For example, some negative control sets like the human proteome are known to contain many false negatives, which can lead to misassignments by the predictor.

**Figure 1:**
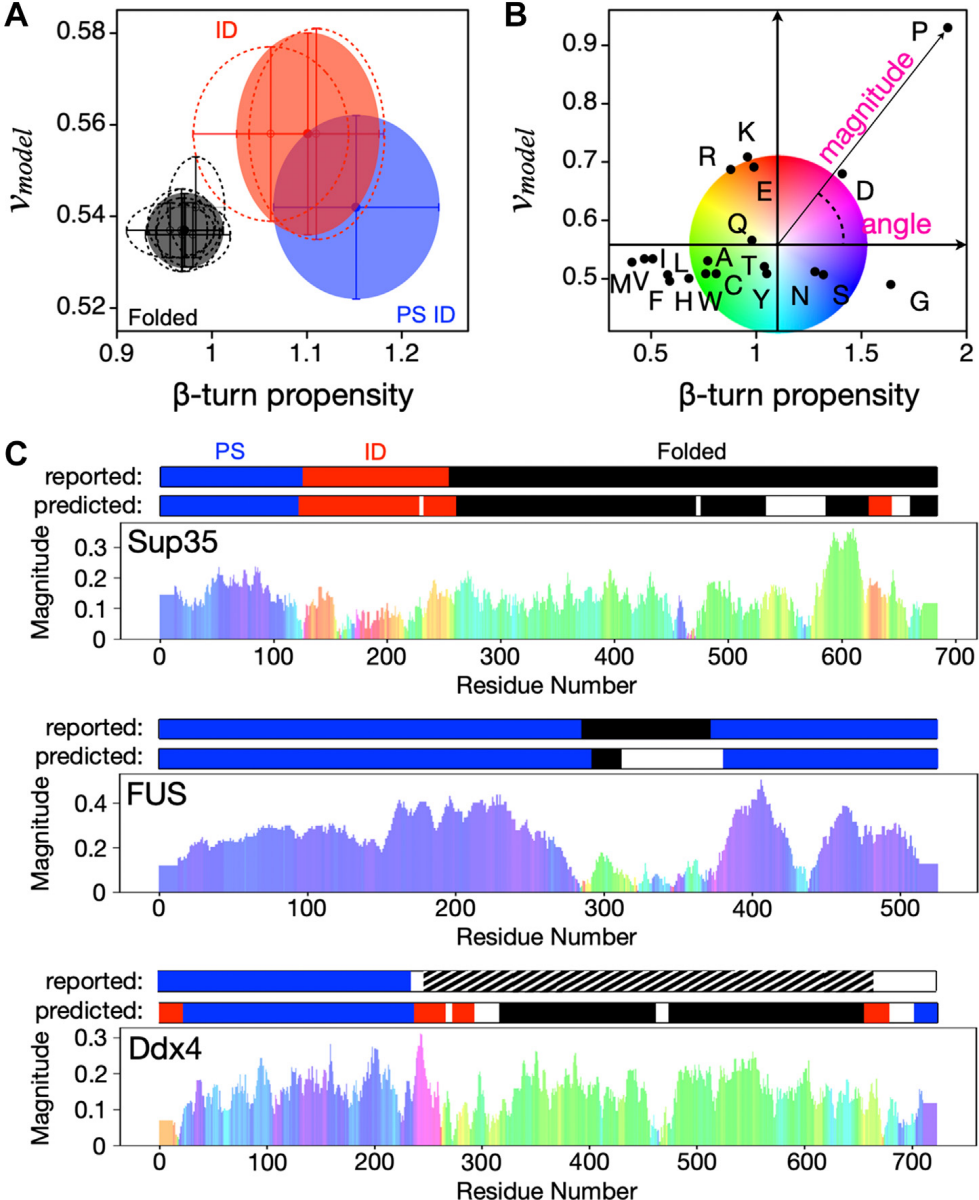
**Sequence-calculated *v***_***model***_**and β-turn propensity separate protein regions by class.***A,* comparing *v*_*model*_ and β-turn propensity in each sequence set. *Filled circles* show the mean and SD in *v*_*model*_ and β-turn propensity in the PS ID (*blue*), ID (*red*), and folded (*black*) sets. *Open* and *dashed circles* show the mean and SD in individual subsets: previous ID and BMRB & DisProt (*red*); previous folded, human, small-to-large, extremophile, membrane, and metamorphic (*black*). *B,* comparing *v*_*model*_ and β-turn propensity in homopolymers (*N* = 100), where amino acid type is identified by its one-letter code. A centralized origin was mapped into this plot at the β-turn propensity and *v*_*model*_ values of 1.101 and 0.558, respectively, which are the means in the ID set. From this origin, every amino acid type can be represented by a distance magnitude and angular displacement, as shown for proline. A color wheel is used to convey angular displacement. *C*, magnitude/color plots are compared to the ParSe (original version) predictions for Sup35 (UniProt ID P05453), FUS (UniProt ID P35637), and Ddx4 (UniProt ID Q9NQI0), and to regions reported (*i.e.*, identified) by experiment. Each figure shows the magnitude (y-axis) and color (angular displacement) by residue number (x-axis), as determined by amino acid type and its magnitude/color from panel B. ParSe predictions use *blue* (PS), *red* (ID), and *black* (folded). *Striped* represents ≥50% identity to a known folded protein. BMRB, Biological Magnetic Resonance Bank; PS, phase-separating; ID, intrinsically disordered.

We first expanded the set of folded proteins. Previously, we selected only folded regions found within known PS proteins. However, this selection may not be justified because it is not known whether folded regions within PS proteins are biased differently in *v*_*model*_ and β-turn propensity compared to folded proteins in general. Subsequently, we expanded the previous folded set (comprised of 82 sequences) to include sequences from 122 human proteins with nonhomologous folded structures (56), 32 proteins with small (*N* = 36) to large (*N* = 415) folded structures (57), 54 folded extremophile proteins (58), 53 folded metamorphic proteins (59), and 90 folded membrane proteins (Table S1). Combined, these folded protein regions represent 421 unique sequences after removing duplicate entries. The folded sets were, overall, similar in both mean *v*_*model*_ (Fig. 1*A* and Table 1) and mean β-turn propensity (Table 2) to the previous folded set obtained from known PS proteins (Tables S2, and S3), indicating that folded regions within PS proteins are indeed similar to folded regions more generally.

**Table 1:** Summary of mean *v*_*model*_ in the protein sequence sets.

Set	Number	*v*_*model*_^a^	*t* test b	*U*-test b
PS ID	224	0.542 ± 0.020	−	−
ID	121	0.558 ± 0.022	2.5e^−10^	1.6e^−11^
Folded	421	0.537 ± 0.008	1.2e^−3^	1.5e^−3^

a Mean ± SD.

b One-tail *p*-value, where *p*-value <0.05 indicates a statistically significant difference in the means of the compared sets. Comparisons are to the PS ID sequence set.

**Table 2:** Summary of mean β-turn propensity in the protein sequence sets

Set	Number	β-Turn propensity a	*t* test b	*U*-test b
PS ID	224	1.152 ± 0.087	−	−
ID	121	1.101 ± 0.075	4.6e^−8^	4.9e^−9^
Folded	421	0.971 ± 0.040	2.0e^−33^	1.1e^−89^

a Mean ± SD.

b One-tail *p*-value, where *p*-value <0.05 indicates a statistically significant difference in the means of the compared sets. Comparisons are to the PS ID sequence set.

Similarly, we expanded the set of IDR sequences not enriched for phase separation potential, called the “ID” set, by adding ID sequences found in the Biological Magnetic Resonance Bank (BMRB) (60) and DisProt (61, 62) databases. NMR experiments are typically performed at relatively high concentrations (≥100 μM), and so BMRB entries that do not explicitly address protein phase separation likely have a low propensity to phase separate. In addition, proteins known to drive phase separation are now annotated in DisProt; therefore, DisProt entries lacking such annotation are at least nominally depleted in phase separation drivers. Moreover, we only selected IDRs from DisProt that were both predicted to be disordered by MetaPredict (63) and were not highly homologous to proteins with folded structures in the Protein Data Bank (PDB) (64) using seqatoms (65). The combined ID set contains 121 unique protein domains (Table S4).

While these expanded datasets show slight differences in mean predicted *v*_*model*_ or β-turn propensity from the datasets used in our previous work (Tables 1, 2, S2, and S3), the expanded sets reinforce our and others’ previous findings that there exist significant differences in *v*_*model*_ (44, 45) or β-turn propensity (53) between the classes of protein regions, in our case, between folded, ID, and PS ID (Fig. 1*A*). These results, as such, confirm that the two sequence-calculated metrics, *v*_*model*_ and β-turn propensity, can be used in combination, as done previously, to predict PS regions within proteins (44).

As our model is the simple summation of contributions from each amino acid, it is useful to consider homopolymers to identify how the amino acid types contribute to each of the three classes of protein regions (Fig. 1*B*). However, because natural PS IDRs are a mixture of amino acids, it is how these amino acids combine that gives a protein its PS properties. For example, homopolymers of Tyr have comparatively low *v*_*model*_ and reside in the “folded” sector of a β-turn propensity *versus v*_*model*_ plot. Tyr also has a higher intrinsic propensity for turn structures than Phe and thus, in a heteropolymer that is sufficiently hydrophilic as to be ID, the presence of Tyr would be more conducive to phase separation than the presence of Phe. More generally, the homopolymer values of *v*_*model*_ and β-turn propensity, when presented in a β-turn propensity *versus v*_*model*_ plot, are consistent with previous characterizations of “order promoting” as compared to “disorder promoting” amino acids (Fig. 1*B*). In particular, we find that homopolymers of Trp, Cys, Phe, Ile, Tyr, Val, Leu, Ala, His, Met, and Thr fall within the “folded” region of the β-turn *versus v*_*model*_ plot, and so are predicted to act as “order promoting” amino acids, while by similar analysis, Arg, Gln, Pro, Glu, Lys, and Asp are “disorder promoting”, and Asn, Ser, and Gly are “phase separation promoting”. This result is similar to conclusions from analyses of protein structures (66, 67), where Trp, Cys, Phe, Ile, Tyr, Val, Leu, and Asn are enriched in folded proteins (“order promoting”), while Ala, Arg, Gln Pro, Glu, Lys, Gly, and Ser are enriched in IDPs (“disorder promoting”), and His, Met, Thr, and Asp are “ambiguous”.

In contrast to previous literature that has focused on the cohesive interactions that drive phase separation, our analysis reveals contributions from both hydrophobic and hydrophilic interactions. In the stickers and spacer model (22, 23), Gly and Ser act as spacers, and so are not thought to drive cohesive interactions that are important for phase separation. However, in our analysis, we find that Asn, Ser, Gly are “phase separation promoting” because they are predicted by our algorithm to promote phase separation relative to both folded and ID. We are not focused on the cohesive interactions themselves, but rather what sequence features are present in proteins that do phase separate. Consistent with previous literature, we hypothesize that both stickers and spacers are required; lacking spacers, a protein would be folded or aggregated, and lacking stickers, a protein is not sufficiently cohesive for phase separation (18, 22, 23, 24, 25).

The clear segregation of some amino acids into the PS ID sector of the β-turn propensity *versus v*_*model*_ plot motivated us to consider whether an approach as simple as color coding of the amino acids would enable identification of PS regions in proteins known to phase separate. Indeed, the phase separation–driving regions of many proteins are visually apparent by our simple visualization tool based on the location of homopolymers in the β-turn propensity *versus v*_*model*_ plot (Fig. 1*B*). The magnitude is related to the propensity and the color indicates the quadrant of the plot; therefore, a shaded bar chart predicts the propensity for a sequence to promote order, disorder, or phase separation. The rapid identification of PS regions in proteins (Fig. 1*C*) such as Ddx4, FUS, and Sup35 (3, 17, 22, 68) led us to conclude that PS regions in proteins are distinctly different than other ID regions. We therefore sought to determine whether these classes of proteins were distinguishable by other amino acid property scales.

### Most amino acid property scales find significant differences between folded, ID, and PS protein regions

We sought to determine if additional sequence-based intrinsic properties were significantly different between protein regions that are folded, ID, or ID with high potential for driving phase separation. To explore this idea, 566 scales of amino acid properties were obtained from the Amino Acid Index Database (54), which is a curated set of numerical indices representing various physicochemical and biochemical properties of the amino acids. This approach is similar to work done to improve coarse-grained models by testing multiple hydrophobicity scales (42). We added to these scales a newly developed hydrophobicity scale designed to predict sequences that drive protein phase separation (19) as well as *v*_*model*_. For each scale and for each sequence, we summed the amino acid scale for amino acids in the sequence and divided by the length, *N*. Welch’s unequal variances *t* test (69), given as a one-tail *p*-value, was used to find scales that show a statistically significant difference in the means of the sequence sets. Using the nonparametric Mann-Whitney *U*-test (70) gave overall similar results (Fig. S1).

**Figure 2:**
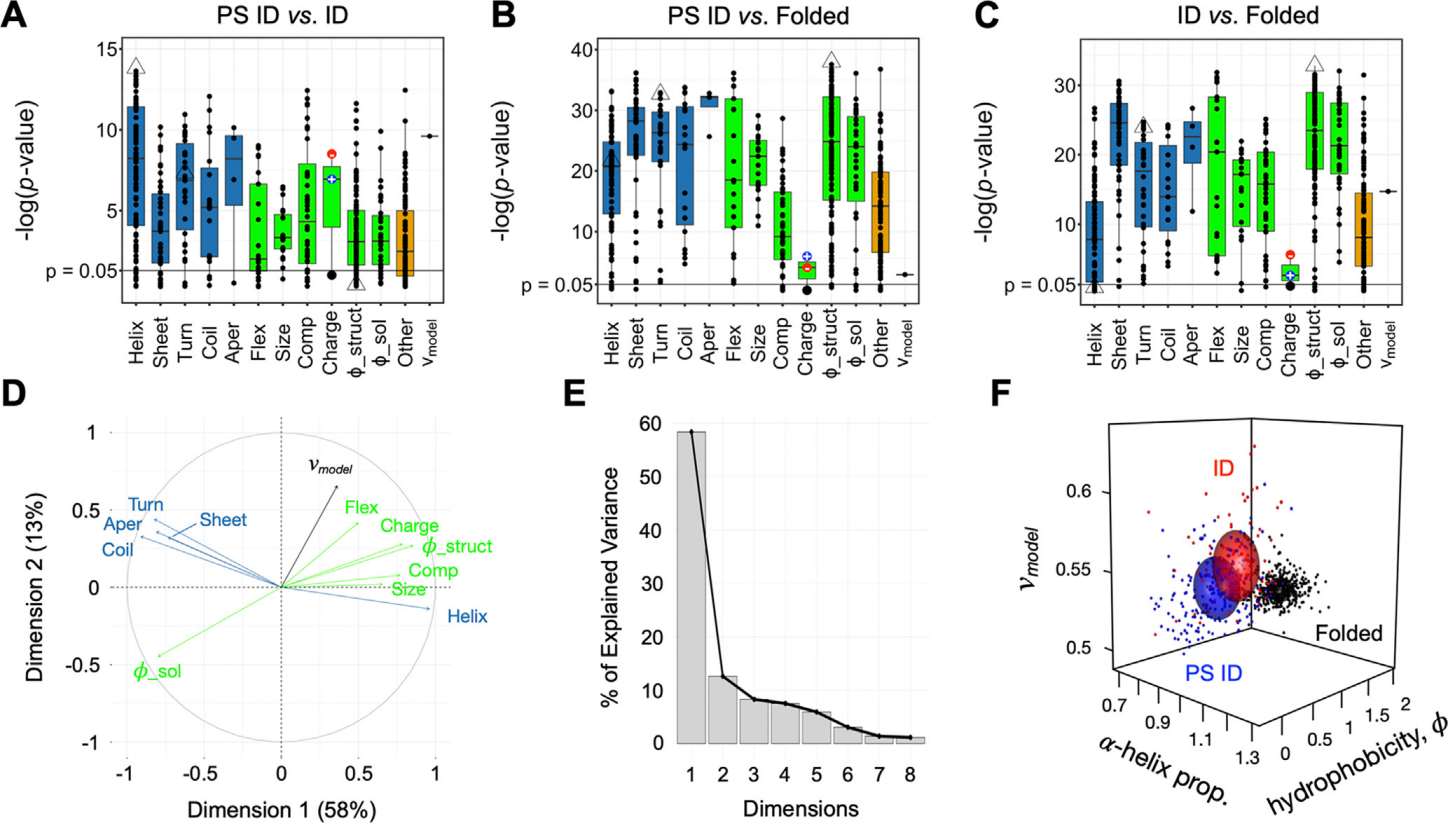
**Robust differences in intrinsic sequence-calculated properties are found when comparing means by protein region class.***A–C*, *p*-values calculated by Welch’s unequal variances *t* test, shown as -log(*p*-value), compares set means in 567 amino acid scales and *v*_*model*_. Conformation-based scales, highlighted by *blue* boxplots, are grouped by type according to α-helix (Helix), sheet or strand (Sheet), β-turn, tight turn, or reverse turn (Turn), coil or loop (Coil), and aperiodic (Aper) propensities. Physicochemical-based scales, highlighted by *green* boxplots, are grouped by type according to flexibility (Flex), size (Size), composition (Comp), negative charge, positive charge, or net charge (Charge), and hydrophobicity (*φ*). Hydrophobicity scales were separated into two types: structure-based (*φ*_struct), where the scale is derived from a structural metric-like burial or contact frequency in surveys of high-resolution protein structures, and solution-based (*φ*_sol), where the scale is obtained from solution studies like measuring the transfer-free energy of the amino acids from water to an organic solvent. Scales (*e.g.*, refractivity, crystal melting point) that did not easily map into a conformation- or physicochemical-based group were combined separately (Other). Boxplots show the dataset median (50th percentile) with the central bar, and the vertical width spans the 25th to 75th percentiles. *Open triangles* highlight the smallest *p*-value when comparing means in the PS ID and ID sets (from an α-helix propensity scale), the smallest *p*-value when comparing means in either the PS ID or ID sets with the folded set (from a structure-based hydrophobicity scale), and the β-turn propensity scale used in ParSe. *D*, bidimensional plot from PCA showing the modes of variance in the combined ID set (PS ID and ID) arising from conformation- (*blue arrows*) and physicochemical-based (*green arrows*) scales relative to the two principal components of variance, given as Dimension 1 and Dimension 2. *E*, scree plot showing the percent of the total variance in the combined set of ID sequences that is captured by each principal component (*i.e.*, dimension). *F*, sequence calculated *v*_*model*_, α-helix propensity, and hydrophobicity for the sequences in the PS ID (*blue*), ID (*red*), and folded (*black*) sets; *spheres* show the set mean ± σ. ID, intrinsically disordered; PS, phase-separating; PCA, principal component analysis.

Figure 2, *A*–*C* show that the different sequence sets have statistically significant different mean values for most scales when compared. For example, 81% of scales give *p*-values <0.05 (indicating means that are different statistically), when comparing ID and PS ID sequences (Fig. 2*A*). Moreover, 13% and 22% of scales yield *p*-values smaller (thus showing a more significant statistical difference) than the *p*-values obtained from *v*_*model*_ and β-turn propensity, respectively, used in ParSe (44). Each scale type (*e.g.*, α-helix propensity, β-turn propensity, hydrophobicity, etc.) had some scales with very low *p*-values and some with *p*-values ≥0.05, suggesting that, overall, most, but not all, conformational- and physicochemical-based scales could substitute for *v*_*model*_ or β-turn propensity in ParSe and likely exhibit some ability for identifying PS IDRs from sequence. This analysis reveals that the physical differences between PS and conventional IDRs are robust across many different scales of amino acid properties (Fig. 2*A*). We conclude that PS regions likely contain a variety of complementary, redundant sequence features that drive phase separation.

The differences between folded and ID (both ID and PS ID) datasets are also robust to different scales of amino acid properties (Fig. 2, *B* and *C*). Ninety-five and ninety-three percent of scales produced *p*-values <0.05 when means were compared between the folded and PS ID, and folded and ID sets, respectively. Almost all amino acid property scales yield statistically significant different means when comparing ID and folded sequences; the best performing scales were based on hydrophobicity. Those hydrophobicity scales with the lowest *p*-values when comparing means in the folded and ID sets had among the highest *p*-values when comparing means in the ID and PS ID sets (and vice versa), consistent with our previous findings that a single metric was insufficient to separate the three datasets.

### PCA identifies two principal modes of variation between proteins

We next sought to determine the degree to which amino acid scales could be combined without significant redundancy when comparing protein sequences. To do so, we used PCA, which characterizes the variability in a dataset (71), in this case, variability arising from different scales being applied to our sequences. Our primary focus is on distinguishing PS IDRs from conventional IDRs because many disordered predictors already exist to separate folded from disordered domains (63, 72, 73). We first selected the scale in each scale type (listed in Fig. 2*A*) with the smallest *p*-value when comparing the ID and PS ID sets, that is, representative scales from each type that are best able to separate ID and PS ID sequences. We additionally included *v*_*model*_, which we found previously to give complementary information to β-turn propensity. Each scale was then used to calculate sequence properties *via* a sliding 25-residue window applied to protein domains in a combined set including both the ID and PS ID datasets or the human proteome. We used a sliding window to avoid averaging properties between regions of proteins with different characteristics (44).

The results of the PCA indicate that most of the variability measured by high-performing scales within these datasets can be captured by 2 to 3 parameters (Figs. 2, *D*, *E* and S2). For both the combined ID dataset including ID and PS ID sequences and the human proteome, approximately 70% of the variability is captured by the first two principal components. Moreover, 58% of the variability in the combined ID set is captured by a single component. The variance arising from conformational propensity scales tend to cluster, as do those with physicochemical metrics like charge, hydrophobicity, and other compositional details. These results are robust to both the number of top-performing scales chosen and to the choice of reference set; we saw similar clustering when we extended this analysis to include the top three performing scales in each type and to the entire human proteome (Fig. S2).

Within these two categories (conformational propensity and physicochemical metrics), high-performing scales function very similarly. As such, the predictive capabilities of amino acid scale combinations within each category are limited. In particular, turn and coil scales applied to protein sequences yield strongly correlated modes of variation that also are mostly anticorrelated with the variance produced from α-helix propensity scales (Figs. 2*D* and S2). In our previous work, we proposed that β-turns could serve as a site for cohesive interactions between protein chains, driving phase separation (44). Our current results, while consistent with this hypothesis, show that this hypothesis cannot easily be distinguished from other structural hypotheses, for example, that coils drive or helix inhibits protein phase separation, because the variation between these scales when applied to our datasets are all highly correlated. In contrast, the variances arising from hydrophobicity, charge, or *v*_*model*_ in our datasets have patterns that, in general, are different from the variances arising from turn, coil, and α-helix conformational propensities.

To illustrate the separation obtained when using complementary top-performing scales, we selected three scales to best separate our three datasets: (1) the top-performing hydrophobicity scale for separating folded from either ID set (from Vendruscolo and coworkers (74)), (2) the top-performing conformational scale in separating ID from PS ID sets, in this case, one predicting α-helical propensity (from Tanaka and Scheraga (75)), and (3) *v*_*model*_ because it was most orthogonal to the latter helix scale in the PCA of our combined ID datasets. As can be seen in Figure 2*F*, significant separation is observed between our different datasets using these three intrinsic sequence properties. In general, the folded domains occupy a region with *φ* >0.08, and the greatest separation between the two disordered sets is observed in the α-helix/*v*_*model*_ plane.

When this approach is used to assess homopolymers of the common amino acids by their placement into a plot of hydrophobicity, α-helix propensity, and *v*_*model*_, the homopolymer results predict that Trp, Cys, Phe, Ile, Tyr, Val, Leu, His, and Met are “order promoting” amino acids, while Ala, Arg, Gln, Pro, Glu, Lys, and Asp are “disorder promoting”, and Asn, Ser, Thr, and Gly are “phase separation promoting” (Fig. S3), similar to what we found previously (Fig. 1*B*). In addition, we can again use this visualization to predict the effect on phase separation of “order promoting” (*i.e.*, hydrophobic) residues when in contexts that are sufficiently hydrophilic as to be ID (Fig. S3*C*). In that context, we find that Tyr promotes phase separation more so than the other hydrophobic amino acids, consistent with previous literature on the importance of Tyr (22, 41).

### Predicting folded, ID, and PS protein regions from sequence

**Figure 3:**
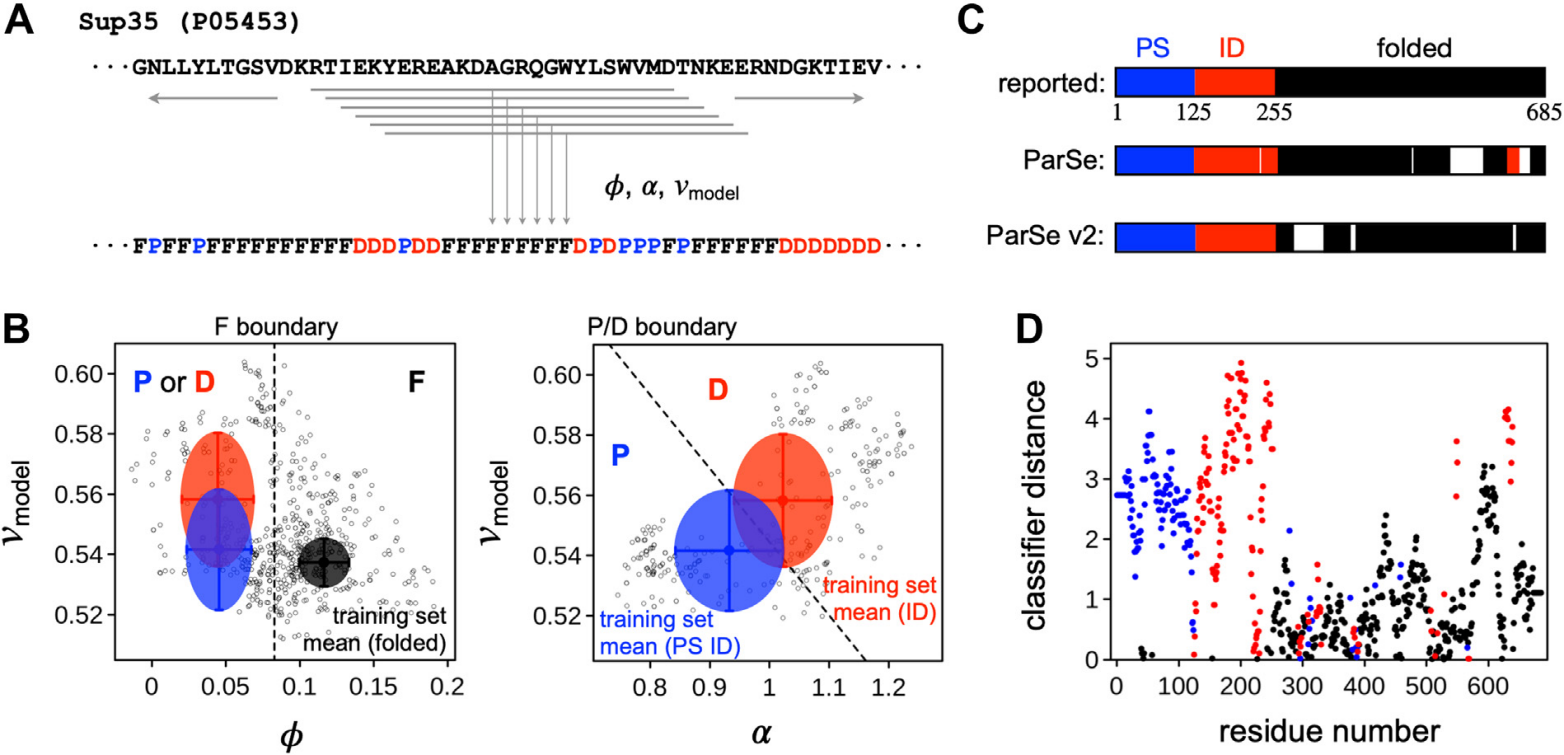
**Predicting protein regions from sequence using the ParSe v2 algorithm.***A*, a sliding window algorithm is used to identify from sequence regions within a protein that match the PS ID, ID, and folded classes. Hydrophobicity (*φ*), α-helix propensity (α), and *v*_*model*_ are calculated for each contiguous stretch of 25-residues, or “window”, in the primary sequence. *B*, each window is assigned a label, F, P, or D, depending on the values of *φ*, α, and *v*_*model*_. In the left figure, *open circles* are *φ* and *v*_*model*_ calculated for each 25-residue window in the Sup35 sequence (UniProt ID P05453); *filled circles* are the mean ± σ in *φ* and *v*_*model*_ in the ID (*red*), PS ID (*blue*), and folded (*black*) sequence sets. Windows with *φ* ≥ the folded set mean - 2σ (*dashed line*) are labeled F. For windows with *φ* < the folded set mean - 2σ, the label is determined by α and *v*_*model*_; P for low α with low *v*_*model*_, or D for high α with high *v*_*model*_, as shown in the right figure. *Filled circles* show the mean ± σ in α and *v*_*model*_ in the ID (*red*) and PS ID (*blue*) sets. *C*, contiguous regions (*N* ≥20) in the Sup35 primary sequence that were 90% of only one label P, D, or F are colored *blue*, *red*, or *black*, respectively, to represent predicted PS, ID, or folded regions. Predictions from the original ParSe and ParSe v2 are compared to the reported regions identified by experiment. *D*, classifier distance of each window assigned to the central residue of the window and then colored according to its label P (*blue*), D (*red*), or F (*black*). ID, intrinsically disordered; PS, phase-separating.

Next, we used the separation obtained from this method to identify protein sequences belonging to folded, ID, or PS ID categories, analogous to what we did for ParSe. Our aim was to see if using these top-performing scales would provide better predictions of PS ID domains. We modified the algorithm making a second-generation version, ParSe version 2 (v2). In this version, as with the original (44), we apply a 25-residue window and then slide this window across a whole sequence in 1-residue steps (Fig. 3*A*) to label individual amino acids as either P (for PS ID), D (for ID), or F (for folded) and then to regions that are at least 90% of any one of these labels (see Experimental procedures, Fig. 3*C*). Both ParSe v1 and v2 accurately delineate regions of Sup35 that have been experimentally determined (68) to behave as ID, PS ID, or folded regions (Fig. 3*C*), and good accuracy is similarly found for other well-studied proteins (3, 17, 22, 76, 77, 78, 79, 80) utilizing diverse reported mechanisms driving protein phase separation (Fig. S4).

**Figure 4:**
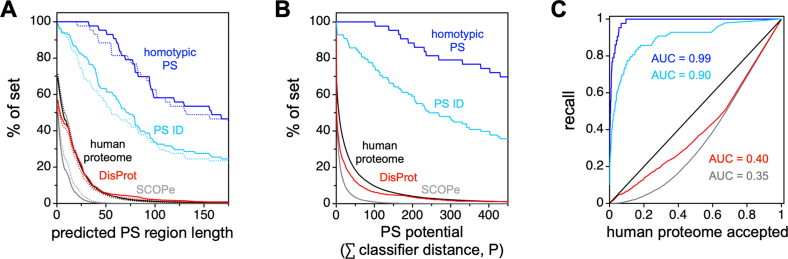
**ParSe-predicted PS regions are rarely found in the human proteome.***A*, ParSe (*stippled lines*) and ParSe v2 (*solid lines*) were used to identify regions in proteins that were ≥90% labeled P, which are referred to as phase-separating, PS, regions. Shown by the y-axis is the percent of proteins in a set with PS regions at least as long as the length indicated by the x-axis. The human proteome (UniProt reference proteome UP000005640) is given by *black lines*; DisProt (minus PS-annotated entries) by *red lines*; SCOPe (version 2.07) by *gray lines*; a set of *in vitro* sufficient homotypic PS proteins by *blue lines*; and the full sequences of the proteins in the PS ID set by *light blue lines*. *B*, the summed P classifier distance was calculated by ParSe v2 for the protein sets in panel *A*. Shown by the y-axis is the percent of proteins in a set with a summed P classifier distance at least as much as the value indicated by the x-axis. *Lines* were colored using the same coloring scheme as in panel *A*. *C*, reproduction of the results in panel B wherein each set was directly compared to the human proteome result. Here, lines show the % of a set (using the same coloring scheme) plotted against the human proteome % of set for values of the summed P classifier distance. ID, intrinsically disordered; PS, phase-separating.

One advantage of our algorithm is that it is very fast and so can easily be applied to large datasets, for example, the human proteome. We measured the prevalence of protein regions predicted by ParSe v2 to have PS potential in the human proteome (Fig. 4) by two methods. First, as previously, we measured the longest predicted region with high PS potential (contiguous regions that are at least 90% labeled P). The results from ParSe v2 are mostly identical to results obtained previously using ParSe (44), whereby only ∼5% of proteins in the human proteome have a predicted P-labeled region that is at least 50 residues in length. Disordered regions taken from DisProt (minus the PS-annotated IDPs) (61, 62) and folded regions taken from SCOPe (Structural Classification of Proteins extended, version 2.07) (81, 82) gave results mirroring the human proteome result in the sense that these sequences are mostly devoid of long regions predicted to have high PS potential. In contrast, the 43 proteins assembled by Vernon *et al* (16) that have been verified *in vitro* to exhibit homotypic phase separation behavior tend to contain long stretches labeled P by ParSe v2, with ∼90% of this set having predicted PS regions ≥50 residues in length. Only ∼63% of the 98 parent proteins from which the PS ID set was derived have predicted PS regions ≥50 residues, wherein not all in this set have been shown to phase separate as purified components.

Second, we developed a numerical score to give a quantitative measure of the confidence of our assignment of P, F, and D labels and to give a single metric to define the PS potential of every protein. Our justification for using a single numerical score is, in part, the dominance of a single principal component in the PCA of the combined ID set (Fig. 2*E*), although we generalized this approach to F-labeled positions as well. In the combined ID datasets, most of the variability was in a single direction nearly orthogonal to the line separating P and D sectors in our plot. As such, we used the linear distance of a 25 amino-acid window into its classifier sector (*i.e.*, F, D, or P sector), relative to the cutoff boundary and normalized by the distance to the boundary of the training set mean (Fig. 3*B*). Values greater than 1 in this *classifier distance* indicate a window located at a distance further from the sector boundary than the distance of the training set mean, whereas values less than 1 indicate a window closer to the cutoff boundary than the training set mean and, as such, possibly with some uncertainty for its classifier label. Classifier distances calculated from the Sup35 sequence are shown in Figure 3*D*, wherein window values have been assigned to the central residue of the window, as we did with the window label.

We used the summed classifier distance for every window labeled P to obtain an overall score for each protein. This method is more robust to situations where multiple smaller regions drive phase separation (Fig. S4), as compared to, for example, Sup35, where a single domain drives phase separation. Windows labeled either F or D do not contribute to this sum. The assumption we are making is that single regions that promote phase separation are sufficient to drive phase separation of larger proteins. This is consistent with the observation that many PS proteins still undergo phase separation when they contain other protein regions or GFP tags (2, 83). As before, we found that only a small fraction of the human proteome consists of proteins with IDRs driving phase separation (44). Indeed, using a cutoff for the summed classifier distance of 100 retains 100% and 76% of the proteins in the Vernon *et al in vitro* sufficient set and the parent proteins of our PS ID set, respectively. In contrast, only 10% of human proteins are predicted to drive phase separation through their IDRs by this cutoff (Fig. 4*B*). Because we are focused solely on IDRs which drive phase separation, excluding multivalent interactions that involve ordered domains, nucleic acids, or other drivers of protein phase separation, the total number of drivers is somewhat larger than this.

We used this whole protein metric (the summed classifier distance of P-labeled windows) to create a recall plot, used to assess prediction performance, for multiple datasets (Figs. 4*C* and S5). The success in recall plots is typically quantified using the area under the curve (AUC), when comparing a test dataset to a comparison dataset (55, 84, 85). Here, in all cases, we used the human proteome as the comparison dataset. The SCOPe database and DisProt (excluding PS-annotated entries) both have AUC values < 0.5 (Fig. 4*C*), indicating that the human proteome does contain more proteins predicted to drive phase separation than these negative control groups. As a result, this approach likely gives a lower bound on the success of a predictor. As expected, our calculated AUC using ParSe v2 is highest on the *in vitro* sufficient phase separation drivers from Vernon *et al* (AUC = 0.99, Fig. 4*C*), which constitute a significant fraction of our positive control dataset (*i.e.*, the parent proteins of the PS ID set). This is likely both because this is the dataset we used for training and because it is also the most highly curated dataset. To further test its efficacy, we measured AUC values for ParSe v2 on datasets of phase separation drivers curated by other groups (16, 55, 84, 85, 86) and found it to perform quite well, with AUC values >0.8 (Fig. S5).

Figures 4*A* and S6 show ParSe v2 is an improvement (*i.e.*, slightly higher recall), albeit marginally, compared to the original ParSe. The strong performance of ParSe v1 is, in part, because even in the original version, we used scales that gave strong separation between datasets. Utilizing scales with weaker predictive value leads to a less efficient predictor, as expected (Fig. S7). A comparison between ParSe v2 and ParSe v1 predictions reveals that the same patterning of P, D, and F regions appears for both predictors (Fig. S8).

We then sought to compare ParSe to other published predictors. Although their data are not as highly curated as others, recent published work by Chen *et al* included predictions from multiple predictors on a publicly available dataset, facilitating comparison to other predictors of protein phase separation (84). Of note, the negative control set in Chen *et al* contains, by our prediction, a higher fraction of IDRs driving phase separation than the human proteome (Fig. S9*D*), although whether this is a problem with the database or with our prediction method is unclear. On their datasets, ParSe performs similarly as measured by AUC scores, to PScore (16), CatGranule (34), and PLAAC (33) in identifying proteins that drive phase separation (Fig. S9, *A*–*C*). The quality of the test one can make of these predictors depends significantly on the quality of the datasets, and so a true test of these predictors will require significantly more experimental data from both positive and negative controls (31, 55).

### Predicting the effects from mutation on phase separation behavior

Despite its simplicity, ParSe can predict the IDR(s) driving phase separation for a wide range of known PS proteins, including FUS, Ddx4, LAF1, and A1. Several of these proteins have been the targets of mutagenesis studies implicating specific interactions between amino acids (*i.e.*, cation-π or cation-anion) in the formation of phase-separated droplets. Cation-π interactions are thought to occur between different amino acids in the chain, and the balance of residues, for example, Arg and Tyr, is thought to be important for phase separation (16, 22, 38). Similarly, net charge per residue, as opposed to simply the number of negative or positive charges (41), as well as the specific charge pattern (27), are also thought to be the key determinants of phase separation.

**Figure 5:**
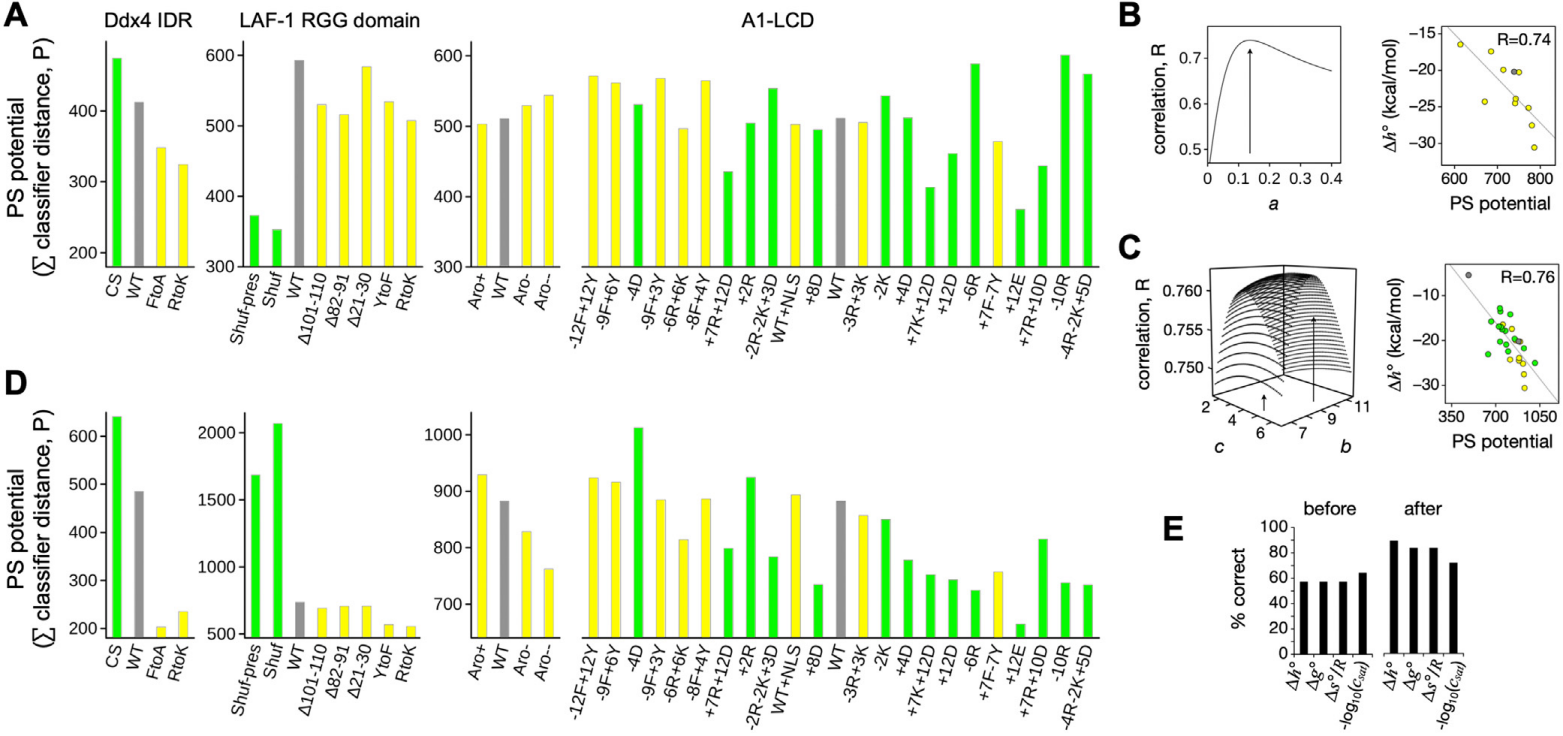
**Predicting mutation effects on phase separation behavior.***A*, the summed classifier distance of P-labeled windows was used to calculate a phase-separating (PS) potential from sequence. Mutants were grouped by experimental study and colored *gray* for WT, *yellow* for mutants with both *NCPR* and *SCD* identical to the WT values, and *green* otherwise (non-WT *NCPR* and *SCD*). Placement left-to-right within a study follows the reported PS potential in rank, from high-to-low, for comparison to the predicted PS potential. A1-LCD mutants used Δ*h°* and not *c*_*sat*_ to establish rank. *B*, A1-LCD mutants with *NCPR* and *SCD* matching the WT values were used to fix *a* in Equation 3 by optimizing the correlation of ParSe-calculated PS potential (including *U*_*π*_) to Δ*h°*; the right figure shows the optimal correlation. *C*, similarly, all A1-LCD and Ddx4 mutants with experimental Δ*h°* were then used to fix *b* and *c* in Equation 4 by optimizing the correlation of ParSe-calculated PS potential (including *U*_*π*_ and *U*_*q*_) to Δ*h°*; the right figure shows the optimal correlation. *D*, ParSe-calculated PS potentials (including *U*_*π*_ and *U*_*q*_ optimized to Δ*h°*) for the mutant and WT sequences. *E*, percent of mutants correctly predicting an increase or decrease in PS potential relative to the WT before and after including *U*_*π*_ and *U*_*q*_ in the calculations. Results are binned according to experimental value that was used to fix *a*, *b*, and *c* in *U*_*π*_ and *U*_*q*_.

Because ParSe is based only on the amino acid composition, and so does not include these higher-order effects involving combinations of amino acid types, we hypothesize that ParSe will have little predictive value for mutations that specifically alter the ratio of these pairwise interactions. More generally, we sought to determine if ParSe v2 could model the effects on phase separation behavior arising from mutations in the protein sequence. We hypothesize that sequence changes targeting P-labeled positions would have the greatest ability to modulate phase separation behavior. To assess this idea, we used the classifier distance whereby a phase separation “potential” was modeled as the summed classifier distance of P-labeled windows in the protein, as we did above in the recall plots. We compared the summed classifier distance with quantitative measures of phase separation behavior from four mutational studies involving three IDRs that individually exhibit phase separation behavior *in vitro* as purified components (3, 18, 27, 41), with sets of published mutations modulating either charge patterning or π-based interactions (Figs. 5 and S10).

As the different studies used different metrics to quantitatively assess phase separation, we first began by simply asking whether the summed classifier distance could accurately reproduce the rank ordering of variants. In Figure 5*A*, we ordered, from left-to-right, in decreasing phase separation “potential” as reported within each individual study the mutant and WT sequences. Shown is the summed classifier distance of P-labeled windows. In the LAF-1 RGG study (27), mutants forming phase-separated droplets at elevated temperatures indicated increased phase separation potential, whereas changes in the saturation concentration, *c*_*sat*_, at a given temperature was used in studies with A1-LCD (18, 41). However, the mutant rank order in *c*_*sat*_ can change with the temperature, caused by differences in the standard molar enthalpy associated with phase separation, Δ*h°*, which reflects the temperature dependence to *c*_*sat*_. To manage this issue, mutant data were separated into two sets. One set corresponding to those mutants with experimental *c*_*sat*_ at 4 °C (Table S6) and a second corresponding to those mutants with experimental Δ*h°*, Δ*s°*, and Δ*g°* (Table S5). Figure 5*A* shows rank order in Δ*h°* for the A1-LCD mutants. Fig. S10 ranks the A1-LCD mutants according to *c*_*sat*_ at 4 °C. The summed classifier distance (*i.e.*, ParSe v2 predicted PS potential) of each mutant trended somewhat with the experimental rank order, correctly predicting an increase or decrease relative to the WT in ∼60% of the mutants as presented in Figure 5 (*i.e.*, with A1-LCD mutants ranked by Δ*h°*) and ∼65% in Fig. S10 (*i.e.*, with A1-LCD mutants ranked by *c*_*sat*_). Thus, ParSe is only moderately able to predict the effects of mutations designed to disrupt pairwise interactions between amino acids such as those arising from aromatic, cation-π, and charge-based interactions. This performance is similar to the performance of PScore, PLAAC, and catGranule (Fig. S11).

To test the importance of pairwise interactions, we explicitly included different types of interactions in our model to try to account for these contributions and possibly improve the trend of calculated potential *versus* observed phase separation behavior. We expanded our calculation of PS potential to include both the summed P classifier distance and terms, quantifying the effects of interactions between amino acids, termed *U*_*π*_ for π-π and cation-π interactions and *U*_*q*_ for charge-based effects. The contribution of these terms toward predicting the effects of mutations can give information on the relative importance of the individual terms. We used *c*_*sat*_, Δ*h*°, Δ*s°*, and Δ*g°* separately to train this calculation, *via* 31 A1-LCD variants with *c*_*sat*_ and 27 A1-LCD and Ddx4 variants with Δ*h*, Δ*s°*, and Δ*g°* (Figs. 5 and S10). As *c*_*sat*_ is highly sensitive to the temperature (41), we expected the thermodynamic properties to be the more reliable metrics of phase separation. Indeed, we were best able to predict the effects of sequence changes on the measured Δ*h°* (Fig. 5*E*). The predicted PS potential combining summed classifier distance with *U*_*π*_ and *U*_*q*_ correctly predicts the directional change relative to WT in ∼90% of the mutants when *U*_*π*_ and *U*_*q*_ were trained against Δ*h*°, and the correlation between experimentally measured Δ*h°* and ParSe-calculated PS potential was reasonably high (R = 0.76; Fig. 5*C*). Thus, explicit consideration of interactions between amino acid types is important for determining PS potential in these mutational studies. It remains to be seen whether ParSe is able to accurately predict PS potential of mutants designed to test other aspects of phase separation, such as its dependence on the presence of partner molecules or on a specific set of solution conditions (*e.g.*, pH, ionic strength, temperature).

Finally, we sought to determine what effect including these corrections to ParSe had on the identification of proteins driving phase separation. Overall, including *U*_*π*_ and *U*_*q*_ into ParSe increases the number of proteins identified that drive phase separation in both the PS sets and the human proteome (Fig. S12). As a result, the AUC when comparing either our PS ID set or the Vernon highly curated set to the human proteome is slightly reduced. However, whether this is a result of correctly classifying more human proteins as driving phase separation or whether we have simply increased the false negative rate remains to be seen.

## Discussion

In this work, we focused on identifying IDRs that drive phase separation, with a particular focus on separating PS IDRs from conventional IDRs that do not drive phase separation. Using carefully curated datasets of ID, PS ID, and folded domains (Figs. 1 and 2), we developed a sequence-based predictor of phase separation (ParSe; Fig. 3) which is fast enough to scan the entire human proteome in minutes on a single computer and as or more accurate than other published predictors in identifying both proteins and regions within proteins that drive phase separation (Figs. 3, S4, S5, S9 and S11). We recognized that a wide variety of amino acid scales show significant differences between the ID and PS ID datasets, indicating that PS IDRs are a robustly different class of protein region than non-PS IDRs (Fig. 2). We conclude that a redundant combination of molecular mechanisms driving cohesive interactions between amino acids is likely at play. This helps to explain why our general predictor of IDR hydrodynamic size (*v*_*model*_) is a strong indicator of PS potential, as we found previously (44). Moreover, by including interactions between amino acids thought to drive phase separation, we were able to match existing data on mutant sequences (Fig. 5). This extension highlights the importance of pair-wise interactions in modulating phase separation.

While our approach has proved very successful, it, like other approaches to this problem, has significant limitations, including limitations in predicting responses to changes in solvent, limitations of the datasets, and limitations of the constraints of the approach chosen. The formation of phase-separated droplets by polymer chains is a result, very generally, of interactions between chains that are stronger than the interactions of the chain with the solvent. As a result, protein phase separation is strongly dependent on the solution environment. Within cells, there are many proteins which assemble into membrane-less organelles only within specific cellular conditions, for example, upon lowering of pH (68). Our results imply that these cellular conditions are such that there are similar sequence features of PS proteins in different biological responses even though the precise solvent conditions may be different. To accurately predict which solution conditions drive phase separation of any individual protein domain would require a detailed understanding of which mechanisms proteins use to drive phase separation, how those mechanisms are modulated by solutions conditions, and how cells modulate solution conditions in different cellular states. As a first step in this process, our aim is to simply improve identification of which IDRs and which potential mechanisms are used by IDRs to drive phase separation in a variety of cellular and solution conditions. Thus, although our predictor has high success in identifying proteins that have been seen experimentally to drive phase separation, we do not yet distinguish between responses to different cellular conditions, or, for example, upper- *versus* lower-critical temperature. The temperature dependence of hydrophobicity scales as used by Dignon *et al* (87) could be a potential future approach to do this. Moreover, IDRs that drive phase separation in very disparate cellular conditions may have unique sequence features and not be identified by ParSe.

A primary limitation of our work, as well as others, is that even our well-curated datasets have misidentified regions. For example, because the IDR in a protein that is responsible for phase separation has not always been identified, we simply used all IDRs from known PS proteins. As a result, our PS ID set likely includes some IDRs which are not involved in phase separation. Similarly, our ID set was curated from proteins that have not yet been identified to phase separate, including those with experiments done at high protein concentration. However, the lack of observation of phase separation at any one experimental condition does not preclude its formation. Indeed, a long history of solution screening for crystallography would indicate that protein behavior can vary dramatically based on solution conditions (88). However, it appears that our PS ID and ID datasets are sufficiently enriched or depleted for PS IDRs for us to identify key properties of IDRs that drive phase separation. For example, the performance of our predictor is improved as the rigor with which the dataset was curated improves. ParSe gives the highest AUC on the dataset from Vernon *et al* containing only those proteins shown to drive homotypic phase separation *in vitro*, compared to datasets containing PS drivers more generally and weaker still on datasets including both PS drivers and proteins recruited to existing droplets (Fig. S5) (16, 84, 85).

Our approach is based primarily on sequence composition and not on sequence patterning or combinations of amino acids. It is surprising how effective this strategy is and how many different scales can be used to distinguish PS IDRs successfully. Nevertheless, our approach, while fast and effective, is unable to identify pairwise protein interactions that may contribute to phase separation. In our analysis of mutants, we introduced a simple potential whereby amino acid pairs are counted, and this clearly improves the ability to predict the effects of mutation on phase separation (Fig. 5). Pairwise interaction patterns are probably better identified by machine learning algorithms or simulation (27, 28, 42, 43, 55, 85). However, the efficacy of our approach appears to indicate that the primary determinant of whether any one sequence will phase separate depends on the overall amino composition, whereas rearrangements, mutations, or posttranslational modifications of that base sequence will modulate that propensity for phase separation. Thus, it appears that the identification of sequences that have the potential to phase separate is an easier problem than identifying how mutation of a few residues will impact that phase separation potential. This result is not specific to our predictor, as none of the predictors tested here showed significantly better correlation with changes in phase separation potential upon mutation (Fig. S11). We additionally note that different experimental measurements of phase separation potential give different ordering of mutants (Figs. 5 and S10), further compounding the issue.

Finally, our approach differs from several others in that we are focused solely on the problem of separating PS IDRs from IDRs that do not phase separate (55, 84). We are thus not able to identify proteins that utilize multivalent interactions between folded domains and other folded, ID, or nucleic acid–binding domains as a primary mechanism for driving phase separation (24, 25, 89, 90). Moreover, we are primarily focused on IDRs that drive phase separation, as opposed to those that are recruited to existing phase-separated droplets, a case which has been recently considered by Chen *et al* (84). Our motivation for this narrow focus is that a broader focus might obscure mechanisms used only by PS IDRs and that interactions between folded domains are, in general, better understood than those between disordered domains.

The strong performance of ParSe on existing datasets, the robust nature of differences between PS IDRs and conventional IDRs, and the high correlation between ParSe and other predictors on databases of PS proteins all give confidence that ParSe is able to identify PS IDRs with significant accuracy. Because of its speed, ParSe can easily be applied to datasets of arbitrarily large size. As an example, we measured the summed classifier distance for the human proteome and found that only a small fraction of the human proteome is likely to drive phase separation (Fig. 4*B*). Moreover, we identified the 500 proteins with the highest summed classifier distance in the human proteome as well as their longest predicted PS IDR (Table S7). Many proteins involved in transcriptional regulation, RNA metabolism, and other functions known to be associated with membrane-less organelles are identified in this process (Fig. S13). However, many proteins are also identified that are not yet associated with a biological process driven by phase separation (*e.g.*, 240 of the 500 cannot be mapped to a gene ontology term (91, 92)). This suggests that, while the fraction of human proteins driving phase separation may be small, not all of the biological processes relying on phase separation have yet been identified.

## Experimental procedures

### Protein databases

A set of 224 IDRs from proteins that exhibit phase separation behavior, used for the PS ID set, was obtained from our prior work (44). For the ID set, we started with 23 IDR sequences used previously (44) and then added all DisProt consensus ID sequences not having the disorder function ontology identifier for phase separation, IDPO:00041 (62). Protein sequences in the BMRB (60) with “disordered” or “IDP” as a keyword or in the entry title were also added to the ID set. BMRB obtained sequences were restricted to those with ≥70% of residue positions classified as disordered by Wishart’s random coil index, using an S^2^ cutoff of 0.6 (93). DisProt and BMRB sequences were culled by Metapredict (63), keeping only those predicted to be ID, and seqatoms (65), excluding those that were highly homologous to folded regions of proteins in the PDB. The folded set started with the 82 folded sequences used previously (44) and then added a set of human proteins with nonhomologous structures (56), proteins with small to large structures (57), extremophile proteins (58), metamorphic proteins (59), and membrane proteins that were found by searching the PDB (64) for the phrase “membrane protein.” Using the PISCES Server (94), the human, extremophile, metamorphic, and membrane proteins had a maximum of 50% sequence identity within each folded subset and only X-ray structures with a resolution better than 2.5 Å.

### Calculation of β-turn propensity and *v*_*model*_

The propensity to form β-turn structures was calculated by ∑ *scale*_*i*_/*N*, where *scale*_*i*_ is the value for amino acid type *i* in the normalized frequencies for β-turn from Levitt (95). The summation is over the protein sequence containing *N* number of amino acids. *v*_*model*_ was introduced previously (44) as a phenomenological substitute to the polymer scaling exponent (51, 52) and used to normalize protein hydrodynamic size to the chain length,

(1) $$v_{model}=\log\left(R_{h}/R_{o}\right)/\log\left(N\right)$$

where *R*_*o*_ is a constant set to 2.16 Å, and the hydrodynamic radius, *R*_*h*_, is calculated from sequence using an equation found to be accurate for monomeric IDPs (49, 50, 96, 97, 98). The equation to calculate *R*_*h*_ for a disordered sequence is,

(2) $$R_{h}=2.16{Å\cdot \;N}^{\left(0.503-0.11\cdot \;\ln\left(f_{PPII}\right)\right)}+0.26\;\cdot \left|Q_{net}\right|-0.29\cdot N^{0.5}$$

where *f*_*PPII*_ is the fractional number of residues in the PPII conformation, and *Q*_*net*_ is the net charge. *f*_*PPII*_ is estimated from ∑ *P*_*PPII,i*_/*N*, where *P*_*PPII,i*_ is the experimental PPII propensity determined for amino acid type *i* in unfolded peptides (99) and the summation is over the protein sequence. *Q*_*net*_ is determined from the number of lysine and arginine residues minus the number of glutamic acid and aspartic acid.

### Principal component analysis

The statistical program R (100) was used to perform PCA on the sequence sets, and the packages ggfortify, ggplot2, factoextra, MetBrewer, and tidyverse were used to render the results. In the PCA, the variables were shifted to be zero centered and scaled to unit variance.

### ParSe v2 algorithm

For an input primary sequence, whereby the amino acids are restricted to the 20 common types, ParSe v2 first reads the sequence to determine its length, *N*. Next, the algorithm uses a sliding window scheme (Fig. 3*A*) to calculate *v*_*model*_, α-helix propensity, and *φ* for every 25-residue segment of the primary sequence. This window scheme can be applied to proteins with *N* >25. *R*_*h*_ is calculated by Equation 2, which in turn is used to determine *v*_*model*_ by Equation 1, by the same method used in the original ParSe described previously (44). α-helix propensity is calculated as the sequence sum divided by *N* using the scale by Tanaka and Scheraga (75). *φ* is calculated as the sequence sum divided by *N* using the hydrophobicity scale by Vendrusculo et al. (74). A window is labeled F if *φ* >0.08 (Fig. 3*B*). If *φ* <0.08, a window is labeled P or D depending on the values of *v*_*model*_ and α-helix propensity. Windows with high α-helix propensity and high *v*_*model*_ are labeled D, while those with low α-helix propensity and low *v*_*model*_ are labeled P. The P/D boundary was determined by the line that bisects the overlapping distributions of *v*_*model*_ and α-helix propensity in the PS ID and ID sets, given by *v*_*model*_ = -0.244⋅α-helix propensity + 0.789. The window label is assigned to the central residue in that window. N- and C-terminal residues not belonging to a central window position are assigned the label of the central residue in the first and last window, respectively, of the whole sequence. Protein regions predicted by ParSe v2 to be PS, ID, or folded are determined by finding contiguous residue positions of length ≥20 that are ≥90% of only one label P, D, or F, respectively. When overlap occurs between adjacent predicted regions, owing to the up to 10% label mixing allowed, this overlap is split evenly between the two adjacent regions.

### Classifier distance calculation

The classifier distance is the normalized distance of a ParSe v2 generated window into its classifier sector (*i.e.*, F, D, or P sector) and relative to the cutoff boundary (Fig. 3*B*). For F-labeled windows, the classifier distance is *φ* (of the window) minus the cutoff value of 0.08 and then normalized to distance of the folded set mean *φ* (0.1164) to the cutoff. Specifically, this is (*φ* – 0.08)/(0.1164–0.08). For P or D labeled windows, first we find the point on the P/D boundary (*v*_*model*_ = -0.244⋅α-helix propensity + 0.789) that makes a perpendicular bisector when paired with the window values of *v*_*model*_ and α-helix propensity. Then the distance between this point and the point defined by the window values of *v*_*model*_ and α-helix propensity is determined. Specifically, this distance is sqrt((α – x)⋅(α – x) + (*v*_*model*_ – y)⋅(*v*_*model*_ – y)), where α is the α-helix propensity, x is (α/0.244 + 0.789 – *v*_*model*_)/(0.244 + 1/0.244), and y is (x – α)/0.244 + *v*_*model*_. This distance is normalized by dividing by 0.019 (the distance from the boundary to either of the set means).

### PSCORE calculation

PSCORE, which is a phase separation propensity predictor (16), was calculated by computer algorithm using the Python script and associated database files available at https://doi.org/10.7554/eLife.31486.022.

### Granule propensity calculation

Granule propensity was calculated by using the catGranule (34) webtool available at http://www.tartaglialab.com.

### PLAAC LLR calculation

LLR score, which identifies prion-containing sequences (101), was calculated by using the webtool available at http://plaac.wi.mit.edu.

### Metapredict calculation

Metapredict score (63), which predicts the presence of ID in a sequence, was calculated by computer algorithm using the Python script available at http://metapredict.net.

### Calculation of *U*_*π*_

The relative contributions of aromatic and cation-π interactions to protein phase separation in our calculations followed the observed rank order by Wang *et al*: Tyr-Arg > Tyr-Lys ∼ Phe-Arg > Phe-Lys (22). To mimic this ranking, we assumed 3:2:1 weighting and, also, that Phe–Tyr interactions would contribute comparably to Phe–Lys interactions,

(3) $$\begin{array}{l} U_{\Pi }=a\cdot \left(3\cdot (\#Y\times \#R/{\left(\#Y-\#R\right)}_{\#Y\neq \#R}\right)\\ +2\cdot \left(\#Y\times \#K/{\left(\#Y-\#k\right)}_{\#Y\neq \#K}\right)+2\cdot \left(\#F\times \#R/{\left(\#F-\#R\right)}_{\#F\neq \#R}\right)\\ +1\cdot \left(\#F\times \#K/{\left(\#F-\#k\right)}_{\#F\neq \#K}\right)+1\cdot \left(\#F\times \#Y/{\left(\#F-\#Y\right)}_{\#F\neq \#Y}\right) \end{array}$$

In Equation 3, #Y, #R, #F, and #K represent the number of Tyr, Arg, Phe, and Lys residues, respectively, in a sequence, calculated on a per-window basis, and *a* is a fitting parameter (see below). Thus, *U*_*π*_ increases with increasing Tyr, Arg, Phe, and Lys content and more so when interaction partners are present at similar levels. When the divisor is zero (*e.g.*, when #Y = #R), it is changed to 1 to avoid infinite potentials.

Window-specific *U*_*π*_ was added to the classifier distance at windows labeled P. Moreover, *U*_*π*_ was applied to D-labeled windows, allowing for the possibility of labels changing from D to P. This would occur when the value for *U*_*π*_ was larger than the classifier distance at a D-labeled window. Thus, protein regions that otherwise have characteristics more like the ID set, in *v*_*model*_ and α-helix propensity, could be labeled P if *U*_*π*_ was large enough. When this occurs, the given classifier distance was determined by the difference between *U*_*π*_ and the original classifier distance of the window formerly labeled D.

The parameter *a* in Equation 3 was determined by finding the optimal correlation of ParSe-calculated PS potential to Δ*h*° (finding *a* = 0.14; Fig. 5*B*), Δ*s*° (finding *a* = 0.08), Δ*g*° (finding *a* = 0.11), or *c*_*sat*_ (finding *a* = 0.28; Fig. S10*B*). In each case, the mutants used to fit *a* were limited to the subset with identical charge and charge patterns, determined by calculating the net charge per residue, *NCPR*, and sequence charge decoration, *SCD*, of each sequence. *NCPR* is the number of Lys and Arg residues minus the number of Glu and Asp residues, divided by *N*. *SCD* is calculated by *N*^-1^∑_*i*_∑_*j,j>i*_(*q*_*i*_*q*_*j*_)|*j-i*|^1/2^, where *q* is the amino acid–specific charge (102).

### Calculation of *U*_*q*_

To model the contributions of charge-based interactions to phase separation, we build upon the observations by Schuster *et al* (27) and Bremer *et al* (41) that changes in *SCD* and *NCPR*, respectively, can affect phase separation potential. Accordingly, a simple charge-based potential was defined,

(4) $$U_{q}=b\cdot SCD+c\cdot \left|NCPR\right|$$

where *b* and *c* are fitting parameters, and *U*_*q*_ is calculated on a per-window basis. *U*_*q*_ is added to the classifier distance at each window labeled P and is applied to windows labeled D, following the scheme described above for *U*_*π*_, again allowing for the possibility of labels changing from D to P. As with *a*, the parameters *b* and *c* were fixed by finding the optimal correlation of calculated PS potential and Δ*h*° (finding 8.4 and 5.6, respectively; Fig. 5*C*), Δ*s*° (finding 4.6 and 7.0, respectively), Δ*g*° (finding 5.2 and 5.4, respectively), or *c*_*sat*_ (finding -16.0 and 33, respectively; Fig. S10*C*).

### Calculation of Δ*h°*, Δ*s°*, and Δ*g°* from temperature dependence to *c*_*sat*_

For some Ddx4 and A1-LCD sequences, Δ*h°* and Δ*s°* (and thus Δ*g°*) were not available, but *c*_*sat*_ measured at different temperatures has been reported (3, 18). For these proteins, the standard molar chemical potential, *μ°*, was used to relate *c*_*sat*_ in the dilute and dense phases, *c*_*dilute*_ and *c*_*dense*_, respectively, to the standard molar enthalpy and entropy associated with phase separation (41),

$$\Delta \mu^{\circ }=\Delta g^{\circ }=\Delta h^{\circ }-T\cdot \Delta s^{\circ }=\mu_{dense}^{\circ }-\mu_{dilute}^{\circ }=\mu_{dense}-R\cdot T\cdot ln\left(c_{dense}/c_{ref}\right)-\left(\mu_{dilute}-R\cdot T\cdot ln\left(c_{dilute}/c_{ref}\right)\right)=R\cdot T\cdot ln(c_{dilute}/c_{dense}),$$

where *μ*_*dense*_ − *μ*_*dilute*_ is zero at equilibrium, *R* is the universal gas constant, and *T* is temperature. By plotting the natural logarithm of *c*_*sat*_ at different temperatures, a linear fit *versus* 1/*T* yields Δ*h°* and Δ*s°*. For A1-LCD mutants, 0.03 *M* was used for *c*_*dense*_ (41). For Ddx4 mutants, 0.01 *M* was used for *c*_*dense*_ (3). Δ*g°* was calculated from Δ*h°* − *T*⋅Δ*s°* and the standard temperature (273.15 K).

### Gene ontology classification

Protein classification by gene ontology was determined by the PANTHER classification system (91, 92) using the webtool available at http:/www.pantherdb.org.

## Data availability

The Parse v2 algorithm written in Fortran, Parse_v2.f, can be downloaded at https://github.com/stevewhitten/ParSe_v2. A webtool version can be used at https://stevewhitten.github.io/Parse_v2_web.

## Supporting information

This article contains supporting information (3, 16, 18, 41, 44, 55, 56, 57, 58, 59, 60, 62, 64, 74, 75, 84, 85, 86, 91, 92, 103, 104, 105, 106).

## Conflict of interest

The authors declare that they have no conflicts of interest with the contents of this article.
